# Therapies for Neovascular Age-Related Macular Degeneration: Current Approaches and Pharmacologic Agents in Development

**DOI:** 10.1155/2013/830837

**Published:** 2013-11-11

**Authors:** Mostafa Hanout, Daniel Ferraz, Mehreen Ansari, Natasha Maqsood, Saleema Kherani, Yasir J. Sepah, Nithya Rajagopalan, Mohamed Ibrahim, Diana V. Do, Quan Dong Nguyen

**Affiliations:** ^1^Retinal Imaging Research and Reading Center, Wilmer Eye Institute, Johns Hopkins University School of Medicine, Baltimore, MD, USA; ^2^Ocular Imaging Research and Reading Center, Stanley M. Truhlsen Eye Institute, University of Nebraska Medical Center, 3902 Leavenworth Street, Omaha, NE, USA; ^3^Department of Ophthalmology, Retina Service, University of São Paulo Medical School, 05403-000 São Paulo, SP, Brazil

## Abstract

As one of the leading causes of blindness, age-related macular degeneration (AMD) has remained at the epicenter of clinical research in ophthalmology. During the past decade, focus of researchers has ranged from understanding the role of vascular endothelial growth factor (VEGF) in the angiogenic cascades to developing new therapies for retinal vascular diseases. Anti-VEGF agents such as ranibizumab and aflibercept are becoming increasingly well-established therapies and have replaced earlier approaches such as laser photocoagulation or photodynamic therapy. Many other new therapeutic agents, which are in the early phase clinical trials, have shown promising results. The purpose of this paper is to briefly review the available treatment modalities for neovascular AMD and then focus on promising new therapies that are currently in various stages of development.

## 1. Introduction

Age-related macular degeneration (AMD) is the leading cause of visual loss in developed countries in individuals over the age of 50 years. Two types of AMD have been reported: nonneovascular (dry AMD) and neovascular (wet AMD). Neovascular AMD is less common, affecting only 10% of AMD patients [1]. However, it is more likely to lead to significant visual loss. Neovascular AMD is characterized by choroidal neovascularization (CNV) development (immature pathological vessels grow from the choroid towards the retina). Leakage from these immature vessels leads to exudation and hemorrhage. Without treatment, the condition causes irreversible damage to the retinal layers and yields central visual loss.

The management of neovascular AMD has markedly changed over the past decade. The approval of pegaptanib sodium (Macugen) in December 2004 by the Food and Drug Administration (FDA) marked the beginning of the molecular era in the treatment of neovascular AMD. Subsequently, the introduction of ranibizumab, bevacizumab, and aflibercept has dramatically changed the treatment paradigm of AMD-related CNV [2]. 

Promising therapeutic molecules continue to emerge and exert their influence through a variety of mechanisms. Some molecules target vascular endothelial growth factor (VEGF), a key player in the disease process, while other molecules have different targets along the angiogenesis cascades.

## 2. Previously Established Therapies

### 2.1. Laser Photocoagulation

Laser photocoagulation works on the principle of cauterizing the feeder vessels of the subfoveal CNV, thus halting subretinal fluid accumulation and preventing progression of the disease [3]. 

The Macular Photocoagulation Study (MPS) compared the usefulness of laser photocoagulation to observation in preventing severe visual loss in patients with neovascular AMD. The study results showed that 60% of nontreated eyes had experienced severe visual loss contrasted to 25% of the treated eyes. This magnitude of benefit observed with laser treatment unjustified withholding of laser treatment from eyes in the observation group and led to early termination of recruitment [3, 4]. 

Combination therapy of laser with other modalities may also lead to potential benefits. However, the incidence of recurrent and persistent CNV after laser treatment decreases the long term effectiveness of this method of therapy [5].

Overall, laser photocoagulation for neovascular AMD may help to slow the progression of vision loss in the long run. However, it may be associated with increased risk of vision loss during the early stage after treatment which lasts for longer durations with subfoveal CNV. Taking this concern into consideration, laser photocoagulation is not recommended with subfoveal CNV, especially with the advent of the several other pharmacologic therapies [6].

### 2.2. Verteporfin (Visudyne, Novartis, Basil, Switzerland)

Photodynamic therapy (PDT), first approved in July 2000 for subfoveal CNV, uses light-activated verteporfin to damage fibrovascular tissue by inducing occlusion of new vessels [7]. The Visudyne in Occult (VIO) study for occult CNV compared the change in best corrected visual acuity (BCVA) from baseline to 12 and 24 months between PDT and placebo. Out of 364 patients with occult CNV, 244 patients were assigned to PDT and 120 patients were assigned to the placebo group. Thirty-seven percent and 47% of patients treated with verteporfin lost 15 letters or more at 12 months and 24 months, respectively, versus 45% and 53% in the placebo group. Verteporfin-treated patients who lost 30 letters or more at these two endpoints were 16% and 24% respectively versus 17% and 25% in the placebo group [8]. 

### 2.3. Antivascular Endothelial Growth Factor

#### 2.3.1. Pegaptanib Sodium (Macugen, EyeTech, New York, NY, USA)

Pegaptanib is a 28-base RNA aptamer that binds selectively and inhibits activation of VEGF-A_165_, which is the most prevalent isoform of VEGF in neovascular AMD [9, 10]. VEGF inhibition Study in Ocular Neovascularization (VISION) was a double-masked, randomized, controlled trial that evaluated three different doses of intravitreal (IVT) pegaptanib sodium for neovascular AMD. A total of 1208 patients were randomized to four groups (who received 0.3 mg, 1.0 mg, and 3.0 mg pegaptanib sodium), respectively, in addition to a sham group. Patients were administered IVT pegaptanib every 6 weeks over a period of 48 weeks. A loss of fewer than 15 letters was observed in 65 to 70% of patients who received pegaptanib (*P* ≤ 0.03) compared to 55% of patients in the sham group at week 54. Severe vision loss of ≥ 30 letters was observed in 8 to 14% of patients who received pegaptanib injection inclusive of all the treatment arms compared to 22% in the sham group. Ocular adverse events (AEs) that occurred more commonly in the study eyes of patients getting IVT pegaptanib included eye pain (34%), vitreous floaters (33%), punctuate keratitis (32%), and increased intraocular pressure (IOP) (20%). The intravitreal injection of pegaptanib was not associated with the potential VEGF inhibition-related systemic AEs recognized with systemic administration of VEGF inhibitors such as hemorrhagic events, thromboembolic events, and vascular hypertensive disorders [9]. 

#### 2.3.2. Ranibizumab (Lucentis; Genentech, Inc., South San Francisco, CA, USA)

Ranibizumab is a recombinant, humanized monoclonal antibody fragment that inhibits all active isoforms of VEGF-A. In the anti-VEGF antibody for the Treatment of Predominantly Classic Choroidal Neovascularization in AMD (ANCHOR) study, which compared PDT with ranibizumab, a mean reduction of 9.8 letters was found in the PDT arm compared to a gain of 8.1 to 10.7 letters in the ranibizumab arm, validating the effectiveness of ranibizumab as a therapy for the improvement of vision in patients with neovascular AMD [11].

In addition to the ANCHOR study, the Minimally Classic/Occult Trial of the anti-VEGF antibody ranibizumab in the Treatment of Neovascular AMD (MARINA) study showed that 94.5% of patients receiving 0.3 mg and 94.6% of patients receiving 0.5 mg dose of ranibizumab lost ≤15 letters at 12 months compared to 62.2% in the sham group. Twenty-five percent of patients receiving 0.3 mg and 33% patients receiving 0.5 mg ranibizumab gained ≥15 letters at 12 months, which remained stable at 24 months as compared to 5.0% in the sham group. There was a mean increase of 6.5 and 7.2 letters in 0.3 mg and 0.5 mg groups, respectively, versus a mean decrease of 10.2 letters in the sham group. The rate of reported endophthalmitis was 1.0% (5 out of 447 patients). Stroke occurred at a rate of 0.8% in sham group, 1.3% in the 0.3 mg group, and 2.5% in the 0.5 mg group [12]. 

 The Safety of Intravitreal Lucentis for AMD (SAILOR) study had two cohorts of patients. Cohort 1 was randomized in a 1 : 1 to receive 3 consecutive monthly injections of 0.3 mg or 0.5 mg ranibizumab then retreated based on visual acuity (VA) and retinal thickness values. Each group was further stratified into treatment naïve and treated patients. Cohort 2 patients received 3 consecutive monthly injections of 0.5 mg ranibizumab and were retreated afterwards at the discretion of the investigator. Treatment-naïve patients gained 5.8 letters with 0.3 mg dose and 7.0 letters with 0.5 mg dose at 3 months. The gain was 0.5 and 2.3 letters for the 0.3 mg and 0.5 mg groups, respectively, at 12 months. For the previously treated patients, 4.6 and 5.8 letter-gain were observed in the 0.3 and 0.5 mg groups respectively at 3 months, whereas 0.3 and 1.7 letter-gain were observed at 12 months. A large number of Cohort 2 patients discontinued from the study, which was noted during data analysis. Overall, the median Snellen VA improved from 20/100 to 20/80 at months 6 and 12. Less than 1% patients in Cohort 1 and 0.1% in Cohort 2 developed endophthalmitis. Among nonocular SAEs, 0.7% and 1.2% patients in 0.3 mg and 0.5 mg ranibizumab group suffered from stroke, respectively, at 12 months [13].

#### 2.3.3. Bevacizumab (Avastin, Genentech, Inc., South San Francisco, CA, USA)

Bevacizumab is a full-length humanized monoclonal antibody that targets all isoforms of VEGF-A. Currently, bevacizumab is the most widely used anti-VEGF agent throughout the world for treatment of neovascular AMD due to its low cost and similar efficacy, with proper treatment schedule [14, 15].

According to the bevacizumab for Neovascular Age-Related Macular Degeneration (ABC trial), there was a statistically significant gain of ≥15 letters among 33% of patients compared to 3% in the standard of care (receiving verteporfin PDT or pegaptanib) and sham groups at the end of one year of study. Two out of 65 patients treated with IVT bevacizumab suffered from intraocular inflammation including iritis, iridocyclitis, vitritis, and anterior chamber inflammation. Systemic SAEs also occurred in 4.6% of patients (1 atrial fibrillation and 2 myocardial infarctions) in the treatment arm [16].

The Comparison of Age-Related Macular Degeneration Treatments Trial (CATT) was a multicenter study that included 1208 patients who were treated with ranibizumab or bevacizumab either monthly or as needed with monthly followup. The results showed that bevacizumab is equivalent to ranibizumab with a visual acuity gain of 8.0 and 8.5 letters, respectively, at one-year followup [17]. The two-year results revealed equivalence (noninferiority) between the two agents in therapeutic benefit and associated adverse effects in favor of monthly treatment over the as-needed regimen. There was a slightly higher number of systemic AEs among patients who were treated with bevacizumab [18].

The interim one-year results of the ranibizumab versus bevacizumab to treat Neovascular Age-Related Macular Degeneration (the IVAN Study), which enrolled 610 patients with treatment-naïve neovascular AMD, showed noninferiority of bevacizumab to ranibizumab in safety and efficacy, both in the monthly and the as-needed regimens [19].

#### 2.3.4. Aflibercept (Eylea, Regeneron, Tarrytown, NY, USA)

Aflibercept (also known as VEGF-Trap) is a human recombinant fusion protein which consists of extracellular domains of VEGF receptor 1 and 2 (VEGFR-1 and -2) fused with Fc portion of IgG1. It binds to VEGF-A, VEGF-B, and placental growth factor (PlGF) [20]. It has a higher affinity for VEGF compared to other anti-VEGFs, including bevacizumab and ranibizumab [21]. 

The Clinical Evaluation of Antiangiogenesis in the Retina Intravitreal Trial 2 (CLEAR-IT 2) is a phase II clinical trial of VEGF Trap-Eye with monthly injection of 0.5 mg or 2 mg aflibercept for the first three months followed by PRN administration from months 4 to 12. Combination of all groups showed a mean gain of VA of 5.7 letters at week 12 and 5.3 letters at week 52 (*P* < 0.0001) [20, 22]. Ocular AEs of aflibercept included conjunctival hemorrhage (38.2%, 5.6%), retinal hemorrhage (14.0%, 11.3%), subjective visual loss (13.4%, 6.3%), vitreous detachment (11.5%, 7.3%), and eye pain (9.6%, 11.4%) at the end of the study [20, 23].

Intravitreal Aflibercept (VEGF Trap-Eye) in Wet Age-Related Macular Degeneration (VIEW 1 and VIEW 2 studies) are two parallel, similarly designed, phase III clinical trials comparing efficacy and safety of aflibercept and ranibizumab for treatment of neovascular AMD. A total of 2419 patients with active subfoveal or juxtafoveal CNV with leakage involving the fovea were enrolled in the two studies. In VIEW 1, patients were recruited from 154 sites in the United States and Canada and in VIEW 2, patients were recruited from 172 sites in Europe, Middle East, Asia, and Latin America. The two studies showed that IVT aflibercept given every 2 months after 3 consecutive monthly injections is equivalent in efficacy and safety to monthly ranibizumab. Thus, aflibercept offers a potential advantage of lessening the burden of monthly evaluations and treatments as well as decreasing the potential risks associated with monthly IVT injections [24]. 

## 3. New Emerging Therapies

### 3.1. Small Interfering RNA (siRNA)

The role of messenger RNA (mRNA) in the production of protein is very crucial. Translation of single mRNA can lead to the production of up to 5,000 copies of a protein molecule. Hence, one can recognize the importance of RNA interference in inhibition of protein assembly by blocking the amplification step in protein synthesis.

siRNA is incorporated into the RNA-Induced Silencing Complex (RISC) inside the cell, enhancing the helicase activity of this complex and leading to selective and potent cleavage and degradation of the mRNA. The siRNA can be engineered to match a specific nucleotide sequence of a target mRNA [25].

1. *siRNA 027 (AGN211745)* is a small interfering RNA that targets VEGFR-1 on the endothelial cells, which in turn mediates new blood vessels formation when stimulated by VEGF or PlGF. IVT injection of siRNA 027 in mice reduced the VEGFR-1 mRNA levels and suppressed the development of CNV at rupture sites of Bruch's membrane as well as decreased retinal neovascularization in oxygen-induced ischemic retinopathy [26, 27]. A dose escalating study of (up to 1600 *μ*g) IVT injection of siRNA in 26 patients showed tolerability and stabilization or improvement of VA in cases with neovascular AMD refractory to other types of treatment [28].
2. *siRNA PF-04523655 (REDD14)* inhibits the expression of hypoxia-induced gene RTP801, which in turn inhibits activation of the mammalian target of rapamycin (mTOR) pathway leading to reduction of VEGF-A production. An additional antiangiogenic effect of mTOR inhibition is achieved by decreasing the response of endothelial cells to VEGF through intracellular signal suppression. In the evaluation of the siRNA PF-04523655 versus ranibizumab for the treatment of neovascular AMD (MONET study), the combination of PF-04523655 with ranibizumab achieved better improvement in BCVA (9.5 letters) than ranibizumab monotherapy group (6.8 letters), which achieved better improvement than PF-04523655 monotherapy (<4 letters), with no safety concerns among the three groups. However, these observations were not statistically significant [29].

### 3.2. Other Antagonists to the VEGF Pathway

#### 3.2.1. Sphingosine-1-phosphate (S1P) Antibody

S1P is a bioactive lipid mediator whose biological activities are moderated via G protein-coupled cell surface receptors that are found on endothelial cells. It is proposed that retinal pigment epithelial cells (RPE) are a major source of S1P in the retina and that the S1P stored and released from RPE is responsible for the pathological angiogenesis, vascular permeability, fibrosis, and inflammatory responses associated with neovascular AMD [30].

Sonepcizumab is a monoclonal antibody that selectively binds to S1P. It was evaluated in mice with oxygen-induced ischemic retinopathy and was found to cause decreased inflow of macrophages into the retina, suppression of retinal neovascularization, and reduced CNV in mice after laser disruption of Bruch's membrane [30]. iSONEP (Lpath, Inc.), the ocular formulation of sonepcizumab, was administered IVT to subjects with CNV secondary to AMD. Results showed regression (>75%) in the CNV lesion in 3 out of 4 patients with occult lesions and resolution of RPE detachment [31]. iSONEP is currently being evaluated in additional clinical trials (clinical trial identifier is NCT01414153) to confirm its efficacy [32].

#### 3.2.2. Squalamine Lactate

Squalamine lactate is an antiangiogenic compound that belongs to the aminosterol class of drugs. It blocks off cell membrane ion transporters that are responsible for keeping cell function in check. Squalamine blocks the action of VEGF and integrin expression when bound to calmodulin, thereby preventing angiogenesis. A phase I/II clinical trial enrolled 40 patients with neovascular AMD and showed vision stability in patients receiving weekly 25 mg/m^2^ and 50 mg/m^2^ of intravenous squalamine lactate (Evizon, Genaera Corporation, Plymouth Meeting, PA) with potential increase in VA in 26% of patients. However, the intravenous formula of the drug was terminated by Genaera and is no longer in clinical development for AMD [31]. A randomized clinical trial evaluating the efficacy and safety of topical squalamine lactate for the treatment of neovascular AMD is currently recruiting patients (clinical trial identifier is NCT01678963) [33].

#### 3.2.3. Palomid 529

Palomid 529 prevents angiogenesis by inhibiting the mammalian target of rapamycin (Akt/mTOR) signal transduction pathway and causing dissociation of rapamycin complexes, TORC1 and TORC2. Animal studies have shown that palomid 529 reduces proliferation and stabilizes the structure of vessels already formed [34]. A phase I trial testing the safety and efficacy of palomid 529 in patients with neovascular AMD who have not responded to anti-VEGF treatments has been completed. However, the results have not yet been published [35]. 

#### 3.2.4. KH902

KH902 is a recombinant, soluble, VEGFR protein in which the binding domains of VEGF receptors 1 and 2 are combined with the Fc portion of IgG. The receptor portion of the molecule has a very high affinity for all VEGF-A isoforms, PlGF 1 and 2, and VEGF-B [36]. It has similar properties as aflibercept. KH902 has been shown to reduce laser-induced choroidal neovascularization in monkeys. In a phase I dose escalating study, 28 patients with neovascular AMD were given a single IVT injection of 0.05 mg, 0.15 mg, 0.5 mg, 1.0 mg, 2.0 mg, and 3.0 mg KH902 and followed up for 12 weeks. No safety concerns were detected and bioactivity of KH902 was suggested with improvement in VA, reduction in central retinal thickness, and reduction in CVN area in patients with exudative AMD [37]. Further studies are being planned.

#### 3.2.5. AAV2-sFLT01

Intravitreal injection of adeno-associated viral (AAV2) vector is being used to deliver an anti-VEGF molecule, sFLT01. AAV2 vectors have minimal toxicity with a potential for long-term expression [38]. Two studies were performed on cynomolgus monkeys to evaluate the efficacy of AAV2-sFLT01. In the first study, the study eye was treated by 2 × 10^8^ or 2 × 10^9^ vector genomes (vg) and the contralateral eye was treated by the same dose of AAV2 vector that does not encode for a transgene (AAV2-Null). Laser-induced CNV occurred 6 weeks later in both groups. None of the treatment eyes demonstrated a statistically significant reduction of CNV leakage compared to the AAV2-Null control eyes. In the second study, dosage was increased to 2 × 10^10^ vg in the study eye with no treatment in the contralateral eye. Laser induced CNV occurred at week 22. Statistically significant reduction of CNV leakage was observed in the treated eyes with only 7% of the treated burns showed leakage compared to 56% of burns in the control eyes. The technology of AAV2-sFLT01 warrants further evaluation to study its potential benefits of long term protection and limited number of injections [39].

#### 3.2.6. MP0112

MP0112 is a designed ankyrin repeat protein (DARPin) based antiangiogenic drug that specifically binds all VEGF-A isoforms. Animal studies indicated a potential of reducing the dosing frequency in humans by 3 to 4 folds compared to current standard therapies [40]. A phase I/II escalating dose multicenter clinical trial investigated the safety and efficacy of intravitreal injection of MP0112 for neovascular AMD over a span of 16 weeks (clinical trial identifier is NCT01086761). However, the study has been terminated by the sponsor after completion of part A of the study and results have not yet been published [41].

### 3.3. Tyrosine Kinase Inhibitors

The biological activity of VEGF is mediated by the VEGFRs. VEGFR-1 and -2 are receptor tyrosine kinases [42]. Therefore, inhibition of tyrosine kinase seems to be a potential therapeutic option for treatment of neovascular AMD.

#### 3.3.1. Vatalanib

Vatalanib is a powerful tyrosine kinase inhibitor with activity against the platelet derived growth factor receptor (PDGFR) and c-Kit receptor kinases. Preclinical studies have demonstrated that this drug prevents angiogenesis [43]. A phase I/II trial to evaluate the safety and efficacy of oral vatalanib combined with PDT has been completed (clinical trial identifier is NCT00138632); the results, however, have not been published yet [44]. 

#### 3.3.2. Pazopanib

Pazopanib is a new generation tyrosine kinase inhibitor against VEGFR, PDGFR, and c-kit [45]. A phase I clinical trial with topical pazopanib in 38 subjects has successfully demonstrated its safety and tolerability. A phase II trial to evaluate its pharmacodynamics, pharmacokinetics, and safety has been completed. However, the results have not been published yet (clinical trial identifier is NCT01072214) [46].

### 3.4. Radiation

 Commonly used anti-VEGF treatments for AMD usually suppress rather than eliminate the disease and usually involve repeated IVT injections. Radiation is a promising and noninvasive therapeutic option that can eliminate the ability of pathologic vascular endothelial cells to replicate, thereby impairing their function and leading to their cellular death. Thus, it may help to decrease the frequency of anti-VEGF IVT injections [47].

#### 3.4.1. IRay

The IRay system is an emerging noninvasive therapeutic option for neovascular AMD. The system delivers 3 overlapping beams through the inferior pars plana to the macula. The spot size of each beam is 3.5 mm at the sclera which gives a 4 mm spot size at the macula. With the overlapping between the 3 beams the effective spot size achieved at the macula is approximately 6 mm. The patient's eligibility for treatment is determined based on the proximity of the optic nerve to the macula [48].

Radiation retinopathy does not usually occur until a dose of 45 Gy is delivered to the retina typically after 2.5 years. Since IRay uses doses of either 16 Gy or 24 Gy, this may decrease the likelihood of radiation retinopathy and makes it a plausible mode of treatment [49]. The IRay Plus Anti-VEGF Treatment for Patients with Neovascular AMD (INTREPID) study is ongoing and aims to evaluate the effectiveness and safety of a one-time radiation therapy with juxtaposed administration of as-needed anti-VEGF injections for treating neovascular AMD [50]. 

#### 3.4.2. Epiretinal Macular Brachytherapy (EMB)

EMB is a new treatment option for neovascular AMD. By allowing the Strontium-90 (Sr90) source to be placed close to the highly diseased area with approximately 5 mm retinal penetration, it enables targeted delivery of radiation to the neovascular tissue [51] and prevent nonocular exposure. Using a 24 Gy source, the required dose can be delivered within 3 to 4 minutes [52]. 

A small scale study yielded positive results for the use of EMB in adjunct with bevacizumab with a mean gain of 8.9 letters in VA at 12 months [53]. However, the Epimacular Brachytherapy for Neovascular Age-related Macular Degeneration (CABERNET) study, a multicenter trial, did not endorse the use of EMB as a monotherapy [47, 54]. The mean VA change at Month 12 compared to baseline for EMB was −0.5 letters with relatively higher incidence of cataract formation and disease progression.

The Macular Epiretinal Brachytherapy in Treated Age-related Macular Degeneration (MERITAGE) study showed relatively favorable results for EMB. Visual improvement was observed in 47.2% of the participants, with decreased required number of anti-VEGF injections to 3 injections only over the 12 month period in more than 50% of participants. Subconjunctival hemorrhages were commonly reported whereas no cases of radiation retinopathy were reported during the course of the study, albeit it was short [55].

Overall, radiation may have potential therapeutic effects; thus far, no study has confirmed its role in the management of neovascular AMD. 

### 3.5. Others

#### 3.5.1. Platelet Derived Growth Factor Antagonists: E10030

In order for newly sprouting vessels to mature and stabilize, endothelial cells need to be coated by pericytes. A key mediator of this process is the platelet-derived growth factor beta (PDGF-*β*) [56]. Inhibitors of PDGF-*β* such as the pegylated aptamer E10030 (Ophthotech) have been demonstrated to prevent this process, thereby making nascent blood vessels susceptible to VEGF inhibition. Safety and pharmacokinetic profile of E10030 have also been evaluated as a monotherapy or in conjunction with ranibizumab in treating neovascular AMD patients [57]. Furthermore, a phase II study to assess the safety and efficacy of E10030 in combination with ranibizumab has been completed (clinical trial identifier is NCT01089517) [58]. Preliminary results showed that combination of E10030 and ranibizumab was superior to ranibizumab monotherapy [59].

## 4. Expert Opinion

The last decade has been a remarkable period for the management of neovascular AMD. Clinician scientists have come far from witnessing patients lose their sights to reviving their vision with new therapies. Since the arrival of photodynamic therapy which, for the first time, stabilized vision, there has been significant advances in the treatment of neovascular AMD with the use of VEGF inhibitors [60, 61]. In the pioneering ANCHOR and MARINA studies, the fluorescein angiography (FA) showed reduction in vascular permeability, leakage, and edema, but the actual structural changes underlying exudative disease remained unaffected by the treatment. The CATT and VIEW studies were able to confirm the effectiveness of VEGF antagonists including bevacizumab, ranibizumab, and aflibercept. A new treatment modality, the antiplatelet derived growth factor (anti-PDGF) agent, has shown the ability to induce neovascular membrane regression and, when used in combination with ranibizumab, produce additional vision gain than anti-VEGF monotherapy. 

There is a consensus on several susceptibility genes for AMD, most notably complement factor H, and LOC387715/ARMS2 [62], which have been shown to affect the patient's response to treatment [63]. Other pharmacological agents and approaches are being evaluated as potential therapies for neovascular AMD as well. These drugs act through different mechanisms, such as modulating the anti-inflammatory response, which may complement current therapies very appropriately. The future of neovascular AMD will very likely depend on our understanding and application of possible combination therapies of different agents and approaches. Further research is warranted in order to achieve this goal and, fortunately, is occurring throughout the world [64].
